# Accurate Detection of Dysmorphic Nuclei Using Dynamic Programming and Supervised Classification

**DOI:** 10.1371/journal.pone.0170688

**Published:** 2017-01-26

**Authors:** Marlies Verschuuren, Jonas De Vylder, Hannes Catrysse, Joke Robijns, Wilfried Philips, Winnok H. De Vos

**Affiliations:** 1 Department of Veterinary Sciences, University of Antwerp, Antwerp, Belgium; 2 Department of Telecommunication and Information Processing, IPI, iMinds, Ghent University, Ghent, Belgium; 3 Institute for Agricultural and Fisheries Research (ILVO), Melle, Belgium; 4 Department of Molecular Biotechnology, Ghent University, Ghent, Belgium; Pennsylvania State Hershey College of Medicine, UNITED STATES

## Abstract

A vast array of pathologies is typified by the presence of nuclei with an abnormal morphology. Dysmorphic nuclear phenotypes feature dramatic size changes or foldings, but also entail much subtler deviations such as nuclear protrusions called blebs. Due to their unpredictable size, shape and intensity, dysmorphic nuclei are often not accurately detected in standard image analysis routines. To enable accurate detection of dysmorphic nuclei in confocal and widefield fluorescence microscopy images, we have developed an automated segmentation algorithm, called Blebbed Nuclei Detector (BleND), which relies on two-pass thresholding for initial nuclear contour detection, and an optimal path finding algorithm, based on dynamic programming, for refining these contours. Using a robust error metric, we show that our method matches manual segmentation in terms of precision and outperforms state-of-the-art nuclear segmentation methods. Its high performance allowed for building and integrating a robust classifier that recognizes dysmorphic nuclei with an accuracy above 95%. The combined segmentation-classification routine is bound to facilitate nucleus-based diagnostics and enable real-time recognition of dysmorphic nuclei in intelligent microscopy workflows.

## Introduction

Nuclear shape changes are present in a broad range of pathologies. Depending on the origin and cell type, nuclei of cancer cells display strikingly different sizes and overt shape alterations such as grooves, folds or lobes, as compared to normal cells [1,2]. Numerous disorders also demonstrate subtler morphological aberrations such as invaginations or protrusions. These protrusions are often referred to as nuclear blebs and they are characteristic for diseases of the nuclear lamina, *i*.*e*., laminopathies [3,4]. In various laminopathies, these blebs represent weak spots, which can sometimes rupture causing illegitimate exchange of nuclear and cytoplasmic proteins [5–8]. Bleb formation has also been observed in viral infections, where it is considered to represent a correlate of nuclear entry and/or egress [9,10]. Despite a clear correlation with disease, not all nuclei in a cell culture display crevices or blebs, and since their formation is time-dependent, it is imperative that they can be automatically detected with high fidelity, preferably in a large number of cells.

In fluorescence microscopy, nuclei are usually labelled using a DNA binding fluorescent dye, which facilitates their segmentation. Many automated nuclear segmentation methods have been described that rely on such a counterstain, including intensity-based [11], active contour [12,13], graph cut [14,15], region growing/merging [16] and dynamic programming-based methods [17,18]. These algorithms often require prior knowledge on the location (dynamic programming), intensity (graph cut) or shape (region merging) of the objects in the image. Unfortunately, dysmorphic nuclei, and more specifically, nuclei with blebs, are typified by subtle shape alterations and lower intensities inside blebs, thereby presenting a difficulty to most existing nuclear segmentation algorithms. To resolve this, we have devised a segmentation method for the detection of dysmorphic nuclei, called BleND (Blebbed Nuclei Detector). It is based on a two-pass thresholding to identify the approximate contours of nuclei, and an optimal path finding algorithm to refine these contours. We have used the algorithm to segment nuclei from a variety of cell types, and we have validated it on a ground truth data set using an integrated error metric. Its high performance allowed for building a robust classifier that accurately discriminates dysmorphic from normal nuclei.

## Methods

### Image data sets

To optimize and benchmark the BleND algorithm, an image data set (widefield microscopy) from DAPI-counterstained human dermal fibroblasts from a compound progeroid syndrome patient (HDF-NCP) was used [19]. This dataset was chosen because it shows high variability in nuclear phenotypes, with both normal and blebbed nuclei being present in the same culture. Additionally, the algorithm was validated with images of other DAPI-counterstained cell types acquired with different imaging modalities: human dermal fibroblasts with a lethal laminopathy phenotype due to a nonsense Y259X homozygous null mutations in the *LMNA* gene (HDF-NULL) [20], which show extremely dysmorphic nuclei, often with an intensity gradient in the nuclear DAPI signal due to chromatin reorganisation (here referred to as chromatin ruffling [21]); human dermal fibroblasts from a Hutchinson—Gilford Progeria syndrome patient (HDF-HGPS, widefield microscopy) [19]; CRISPR/CAS9-genome edited *ZMPSTE24* knockout HeLa cells (HeLa-ZKO; point scanning confocal microscopy); genome-edited *LMNA* knockout human HT-1080 fibrosarcoma cells (HT-LKO, widefield microscopy) [8]; mouse primary hippocampal neurons (spinning disk confocal microscopy) [22].

Widefield images were acquired using a Nikon Ti fluorescence microscope equipped with an Andor DU-885 X-266 camera. Point scanning confocal images were acquired with a Nikon A1R system and spinning disk confocal images were acquired with a Perkin Elmer Ultraview system both mounted on a Nikon Ti microscope. Acquisitions were performed using either a 40x dry (NA = 1.0) objective, 40x oil (Plan Apo, NA = 1.30) objective, or 60x oil objective (Plan Apo VC, NA = 1.40).

### Image processing

**Fig 1:**
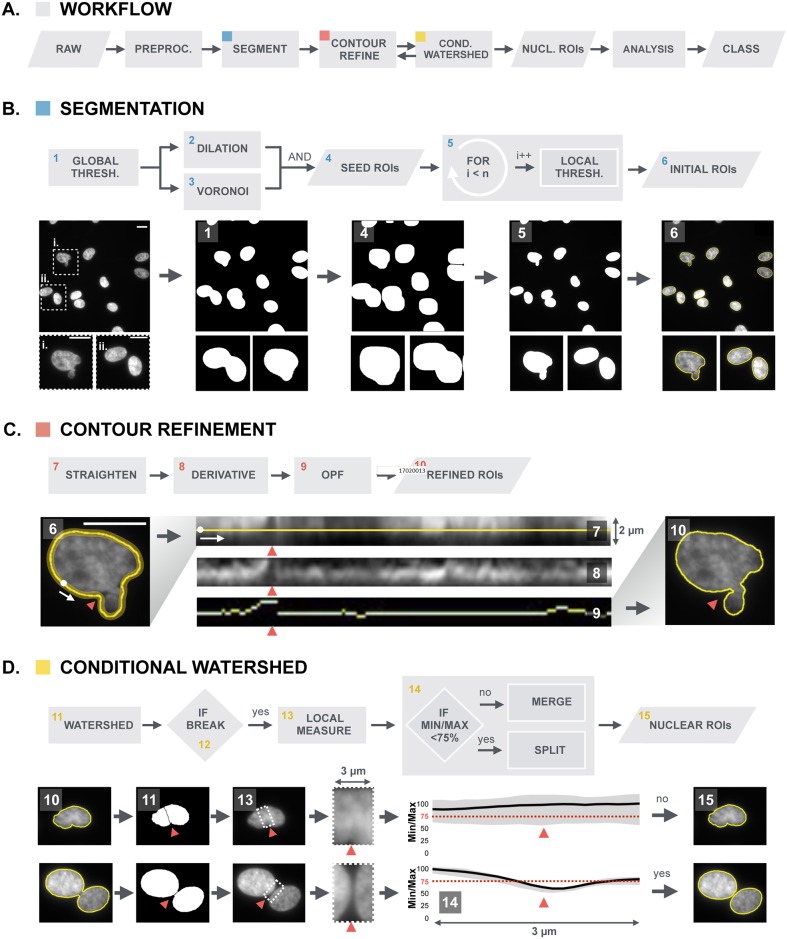
Overview of the BleND segmentation algorithm. (A) Workflow of the algorithm built on (B) intensity-based segmentation, (C) contour refinement, and (D) conditional watershed; (B) The segmentation process is implemented as a two-pass thresholding algorithm that generates “initial ROIs” of nuclei in the preprocessed image (i: dysmorphic nucleus, ii: two juxtaposed normal nuclei). A global thresholding is performed on the image, which creates a binary mask (1). The objects identified herein are dilated by 3 μm and combined (Boolean AND operation) with a Voronoi tessellation mask to ascertain that the dilated objects do not fuse. For each resulting “seed ROI” (4), a local threshold (5) is determined yielding an initial nuclear ROI (6) that is more accurate than the seed ROI (note the improved segmentation for the dysmorphic and juxtaposed nuclei); (C) In the subsequent contour refinement procedure, the initial ROI is used (6) to straighten a 2μm wide region along the nuclear periphery (white dot indicates the point where the contour was opened and the white arrow indicates the direction of the straightening) (7). In this rectangular representation, the edge of the nucleus is enhanced by convolution with a vertical Sobel kernel (8). Then, an optimal path finding (OPF) algorithm determines the path with the highest path strength (9). The OPF algorithm effectively detects crevices surrounding nuclear blebs (red arrowhead). The contour of the nucleus is then reconstructed to generate a “refined ROI” and this process is repeated until the optimal path no longer changes (10); (D) To segment neighboring nuclei that could not be separated in the previous steps, a conditional watershed was implemented in which correct and incorrect splits were discriminated based on a size criterion and an intensity drop along the separation line (red arrowhead). This intensity drop is calculated as a median intensity profile perpendicular to the separation line (13). The user defines a threshold for the acquired intensity drop. In this example, the threshold is set at 0.75. If there is an intensity drop in the median profile of less than 25%—Min/Max intensity ratio above the 75% (dotted red) line (14)—the split is regarded as incorrect and the two parts of the nucleus are merged (15). If the drop is bigger, the split is regarded as being correct and it is retained to generate new nuclear ROIs.

BleND was implemented as a Java plugin in the image processing software FIJI [23], a packaged version of ImageJ [24], and is freely available at https://github.com/VerschuurenM/BLEND. The general pipeline is depicted in Fig 1A. In brief, after pre-processing, an intensity-based segmentation (two-pass thresholding) of the pre-processed image allows identifying initial nuclear regions of interest (ROIs) in the image and generates contours that are refined using the contour refinement algorithm. Subsequently, adjacent nuclei are split using a conditional watershed algorithm. The contours (ROIs) that are newly generated in this process, will again be refined using the same contour refinement algorithm. The separate steps are described in more detail below.

#### Pre-processing

Background subtraction and multiple standard available linear and non-linear image filters (Gaussian, Median, Mean, Minimum, Maximum and Variance) are implemented in BleND; the scale of which can be defined by the user. This allows correcting for imperfect illumination, noise and intranuclear intensity variations (*e*.*g*., chromocenters in mouse nuclei).

#### Segmentation

Since not all nuclei have the same average intensity, a global threshold can under- or overestimate their boundaries. In addition, blebs can have significantly lower intensities, causing them to become falsely assigned to the background (Fig 1B, inset i). To account for this problem, a two-pass thresholding was integrated that performs a rough global (image-based) thresholding, followed by a local (region-based) thresholding. Global thresholding serves to estimate the approximate location of all nuclei, whether they are clustered or not. The result is a set of “seed ROIs”. These seed ROIs are conditionally dilated by maximally 3 μm, with their expansion being restricted by boundaries defined via Voronoi tessellation on the same seed ROIs. This prevents neighbouring regions from merging during the dilation process. Next, a local threshold is calculated within the conditionally dilated seed ROIs so as to obtain a better delineation of the actual contours (Fig 1B, inset i) and separation of neighbouring nuclei (Fig 1B, inset ii). The end result of two-pass thresholding is a set of “initial ROIs” for individual nuclei. All the automatic threshold algorithms that are implemented in FIJI were assessed for global and local thresholding (Huang [25], Intermodes [26], (IJ_)Isodata [27], Li [28], Maximum entropy [29], Mean [30], Minimum error [31], Minimum [26], Moment preserving [32], Otsu [33], Percentile [34], RenyiEntropy [29], Shanbhag [35], Triangle [36], and Yen [37]).

#### Contour refinement

**Fig 2:**
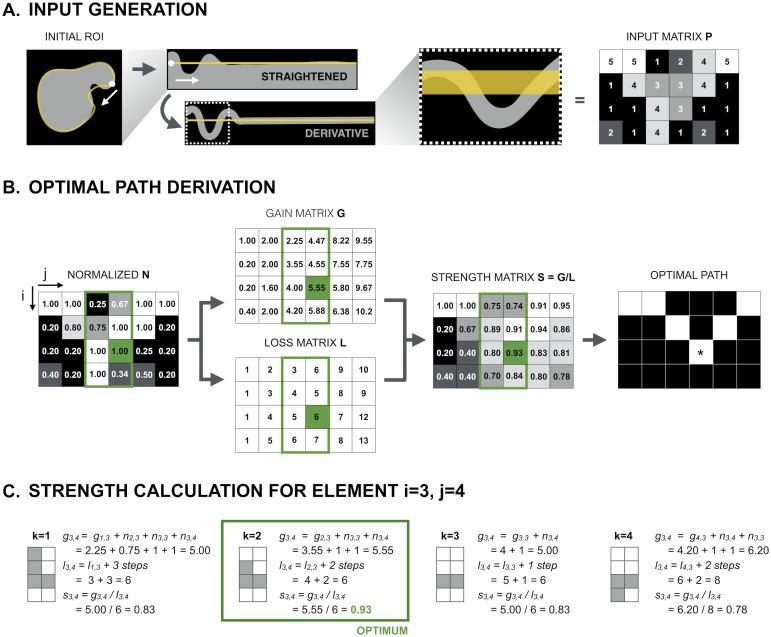
Numerical example of the optimal path finding algorithm. (A) Schematic representation of the generation of input matrix **P**. The vertical derivative of the straightened representation of the nuclear periphery—defined as 2μm wide band surrounding the initial ROI—serves as input matrix **P** for the optimal path finding algorithm (OPF); (B) Columns of **P** are first normalized (divided by the resp. maximum) after which the optimal path is calculated on the normalized matrix **N** using a dynamic programming approach. Starting from the left side of the matrix, a strength function *s*_*i*,*j*_ (strength matrix **S**) is calculated for every matrix element *n*_*i*,*j*_ that takes the ratio of the sum of the intensity along the path (*g*_*i*,*j*_; gain matrix **G**) and the total path length (*l*_*i*,*j*_; loss matrix **L**). The optimal path in **S** is defined by the elements with the highest value per column; (C) Optimal path calculation for the element on row 3 and column 4 of matrix **N** (*n*_*3*,*4*_, marked green in B). The algorithm calculates the gain (*g*_*3*,*4*_), loss (*l*_*3*,*4*_) and strength (*s*_*3*,*4*_ = *g*_*3*,*4*_*/l*_*3*,*4*_) for all 4 possible paths starting in the previous column—in this case column 3—to element *n*_*3*,*4*_. Since the algorithm is progressive, the values for gain, loss and strength have already been calculated for all elements in column 3. Thus, for element *n*_*3*,*4*_, calculation of the gain comes down to summing the value of the first element *k* of the path in the gain matrix (*g*_*k*,*3*_, the total gain up to that point) with the values of **N** along the rest of the path. Likewise, the loss for element *n*_*3*,*4*_ is calculated by summing the value of the first element *k* of the path in the loss matrix (*l*_*k*,*3*_, the total loss up to that point) with the total number of steps to *n*_*3*,*4*_.

**Fig 3:**
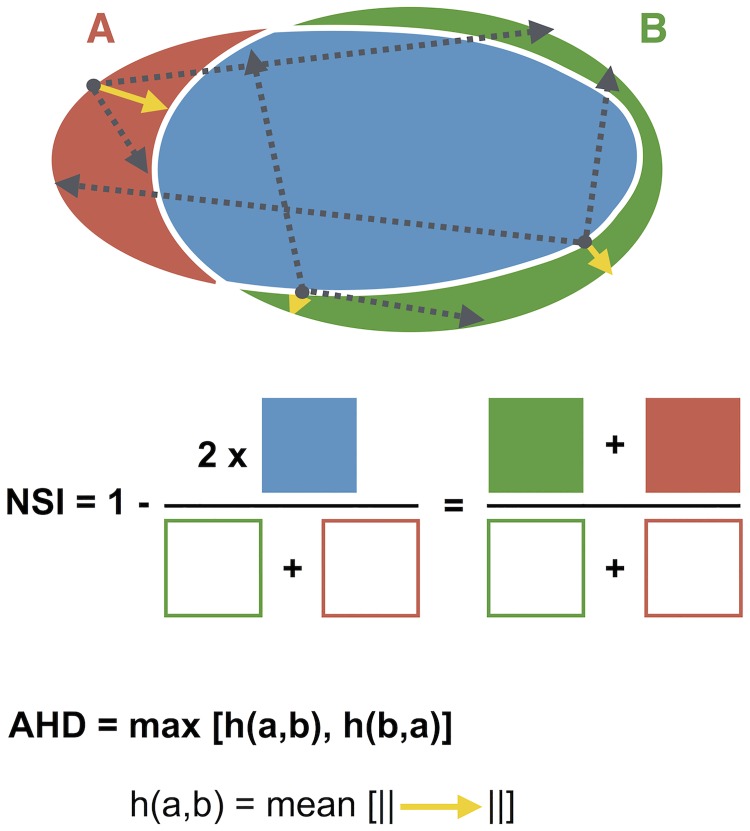
Schematic representation of the error metrics used for validation of the segmentation algorithm. Individual BleND segmentations (red line) were compared to the respective GTs (green line) using two error metrics: the average Hausdorff distance (AHD) and a non-similarity index (NSI). The AHD is calculated as the average of the minimal distances (yellow arrows)–selected among all possible distances (examples in dotted grey arrows)–between the pixels of both contours. The NSI is derived as the non-overlapping area (red and green area) divided by the sum of the total area described by these contours (red and green line).

**Fig 4:**
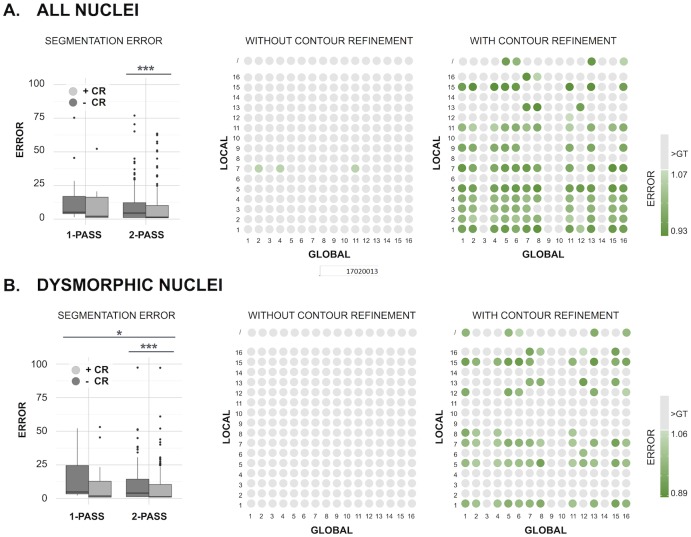
Comparison of performance errors for dysmorphic nuclei. Boxplots and dot plots of the performance errors of all threshold combinations using 1-pass (only global) or 2-pass thresholding (global and local), with (+CR) or without (-CR) contour refinement for (A) all nuclei, and (B) for dysmorphic nuclei only. Asterisks mark statistically significant differences according to the Wilcoxon rank-sum test (one sided) (* P < 0.05, *** P < 0.005). The outliers in the boxplots represent inadequate segmentations caused by an error-prone thresholding method. The color in the dot plots represents the error, with values falling within the error range of the ground truth (GT) comparisons displayed in green hues, and values exceeding this range in light grey. The numbers on the axes of the dot plots represent different threshold methods: 1: Huang, 2: Intermodes, 3: IJ_Isodata, 4: Isodata, 5: Li, 6: Maximum entropy, 7: Mean, 8: Minimum error, 9: Minimum, 10: Moment preserving, 11: Otsu, 12: Percentile, 13: RenyiEntropy, 14: Shanbhag, 15: Triangle, 16: Yen.

**Fig 5:**
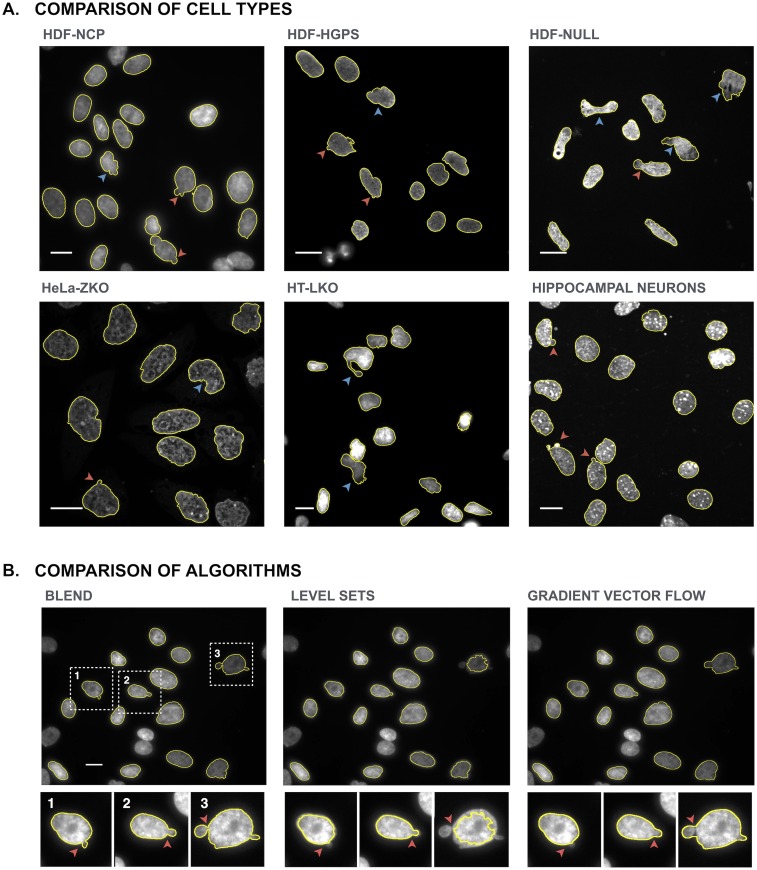
Segmentation results for different cell types and contour refinement algorithms. (A) Segmentation results for DAPI counterstained nuclei of HDF-NCP (widefield microscopy), HDF-HGPS (widefield microscopy), HDF-NULL (widefield microscopy), HeLa-ZKO (point scanning confocal microscopy), HT-LKO (widefield microscopy) cells and mouse primary hippocampal neurons (spinning disk confocal microscopy). Blebbed (red arrowheads) and/or severely deformed nuclei (blue arrowheads) are accurately delineated; (B) Comparison of BleND with level set active contour and gradient vector flow active contour algorithms on an image of HDF-NCP cells. Insets show contrast-stretched, magnified views of selected regions. Due to locally weaker signals, blebs (red arrowheads) are poorly detected with the level set active contour algorithm. The gradient vector flow algorithm performs better, but fails to detect subtler blebs (region 1) and does not accurately delineate deep crevices surrounding blebs (regions 2,3).

**Fig 6:**
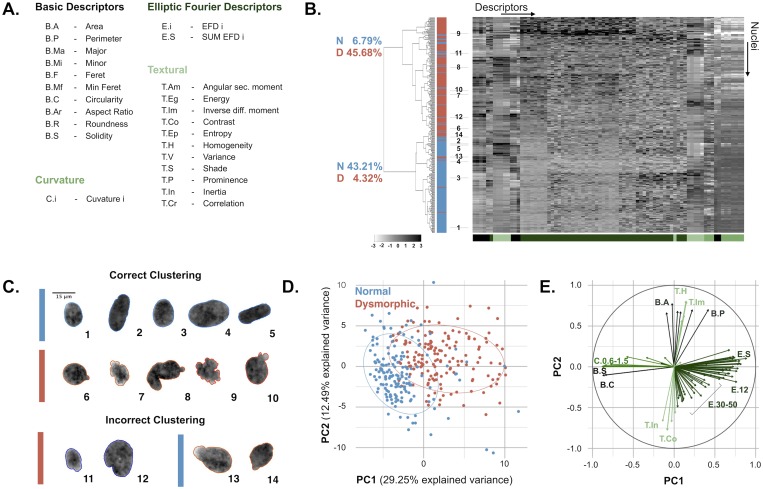
Unsupervised classification of automatically segmented nuclei. (A) Overview of the morpho-textural feature set that was extracted from 324 segmented nuclei; (B) Heatmap representing the grayscale-coded z-scores of all the features (columns) for all individual nuclei (rows). Hierarchical clustering on this dataset largely, but not completely, separates normal (blue) from dysmorphic (red) nuclei populations as indicated by the dendrogram on the left. (C) Example nuclei that have been correctly or incorrectly clustered. Colored outlines represent the manually assigned class, whereas the colored bar represents the assigned class by clustering (blue: normal and red: dysmorphic nuclei). Numbers link segmentations of selected nuclei to their position in the heatmap; (D) Principal component analysis of the data set yields two distinct but not fully separated clusters for the two classes as illustrated by a bi-plot explaining 42% of the variance. Discrimination of the two groups is predominantly in the direction of PC1; (E) The factor map reveals correlated features in PC space.

**Fig 7:**
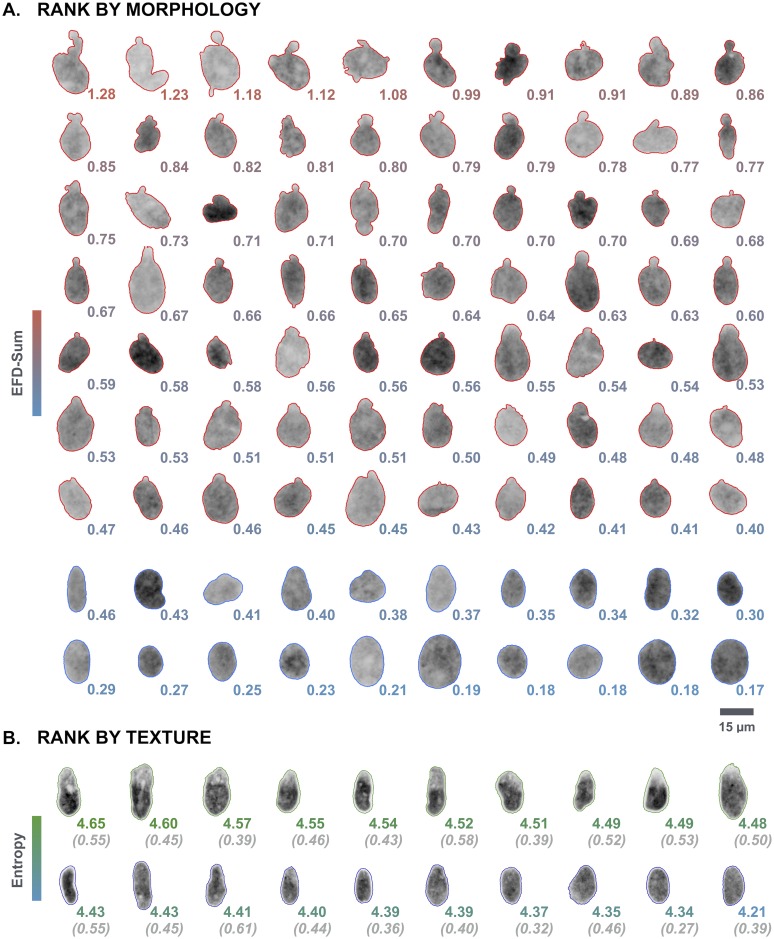
Discrimination of dysmorphic nuclei based on elliptic Fourier descriptors. (A) Morphology-based ranking of HDF-NCP nuclei. Both dysmorphic (red) and normal (blue) nuclei of HDF-NCP are ranked according to their summed EFD value (color coded). Severely deformed nuclei have higher EFD values than nuclei with small blebs, which in turn have larger EFD values than regular, ovoid-shaped nuclei. (B) Texture-based ranking for HDF-NULL cells. Dysmorphic nuclei are characterized by an intensity gradient due to an chromatin ruffling. Normal and aberrant nuclei of comparable shape (EFD value in italic and in brackets) can be distinguished based on the value of the entropy texture parameter (color coded).

After two-pass thresholding, crevices and invaginations surrounding blebs are not yet accurately delineated. In order to improve the initial ROIs, a contour refinement step was implemented (Fig 1C), which relies on contour straightening, a directional derivative and an optimal path finding algorithm. First, the boundary of the nucleus is straightened using an algorithm based on two-dimensional cubic splines [38], thereby generating a rectangular representation of a 2μm-wide region (1μm in both directions) surrounding the initial ROI (Fig 1C-7). Next, the edge of the nucleus is specifically enhanced by calculating the vertical derivative of the straightened image (Fig 1C-8). Finally, the exact contour is determined on the derivative image using an optimal path finding (OPF) algorithm (Fig 1C-9). Among all possible paths that can be drawn from left to right, the optimal path is found by maximizing the mean intensity of the path, defined as the ratio of the total intensity of the path (gain) to the total path length (loss). We refer to this parameter as the “path strength”. The underlying assumption is that the edge response (*i*.*e*., the intensity of the derivative) will be the strongest at the true boundary of the nucleus. However, to prevent intranuclear intensity fluctuations or debris (also having a strong edge response) from skewing the boundary detection, a penalty is introduced for the total distance of the calculated path.

A numerical example of the OPF is represented in Fig 2. The derivative of the straightened image serves as input matrix **P** with dimensions (*q*, *r*), for the OPF algorithm (Fig 2A). The columns of the input matrix **P** are first divided by the column maxima (yielding normalized matrix **N**) to account for any declines in intensity that might occur in blebs or invaginations, so that they have an equal contribution to the average path strength. The optimal path is then calculated on the normalized matrix **N** using a dynamic programming approach. Starting from the left side of matrix **N**, the strength matrix **S**, gain matrix **G** and loss matrix **L** are simultaneously calculated (Fig 2B). Individual elements of each matrix (respectively *s*_*i*,*j*_, *g*_*i*,*j*_ and *l*_*i*,*j*_) are recursively determined per column according to the strength function displayed in Eq 1. For all possible paths to element *n*_*i*,*j*_ of **N**, a value for *s* is calculated, only to retain the path that provides the maximal path strength *s*_*i*,*j*_ (Fig 2C). Since the elements of the preceding columns have already been determined, this procedure boils down to finding the optimal node (element with row index *d*) in the *q* rows of the former column (with index *j-1*). The corresponding gain (*g*_*i*,*j*_) is determined by summing the value of this node (*g*_*d*,*j-1*_) in matrix **G**, with the values of the elements of matrix **N** that lie in between *n*_*d*,*j-1*_ and *n*_*i*,*j*_. The corresponding loss (*l*_*i*,*j*_) is determined by summing the value of this node (*l*_*d*,*j-1*_) in matrix **L** with the number of matrix elements that lie in between *n*_*d*,*j-1*_ and *n*_*i*,*j*_ (|*n*_*x*,*j-1*_*|* + 1 with x an element of [i,d[).

$$\{\begin{array}{c} s\left(N,G,L,i,j,k\right)=\frac{g_{k,j-1}+\;\sum_{x}\;n_{x,j-1}+\;n_{i,j}}{l_{k,j-1}+\left|n_{x,j-1}\right|+1}\;\;\;\;\;\;with\;x\in [\text{i},\text{k}[\\ d={\text{argmax}}_{k\in \left[1,q\right]}[s\left(N,G,L,i,j,k\right)]\\ s_{i,j}=\frac{g_{i,j}}{l_{i,j}}=s\left(N,G,L,i,j,d\right)\\ l_{i,j}=l_{d,j-1}+\;\left|n_{x,j-1}\right|+1\;\;\;\;\;\;with\;x\in [\text{i},\text{d}[\\ g_{i,j}=s\left(N,G,L,i,j,d\right)\;\cdot l_{i,j}=\;g_{d,j-1}+\;\sum_{x}\;n_{x,j-1}+\;n_{i,j}\;\;\;\;\;\;\;with\;x\in [\text{i},\text{d}[ \end{array}$$ (1)

The optimal path is defined as the elements in each column of **S** with the highest value, allowing propagation angles up to 90° to accurately describe crevices surrounding blebs; this is in contrast with previously described methods where only angles < 45° were allowed [17,18] (Fig 1C-9). Once the optimal path is found on the derivative of the straightened image, it is converted to a closed contour, yielding the “refined ROI” for that specific nucleus (Fig 1C-10). The OPF is iteratively applied, each time using the newly created contour as substrate, for a defined number of cycles, to enable the detection of crevices that are bigger than 1μm, the half width of the rectangular region.

#### Conditional watershed

To separate clustered nuclei but prevent small structures like blebs from being disconnected, the watershed algorithm [39] was modified with two criteria for merging objects that were split incorrectly. The first criterion is based on object size: separate objects should not have an area below the minimal size assigned by the user. The second criterion is based on the presence of a sufficiently strong intensity decay (background signal) between adjacent nuclei, assuming that incorrectly split nuclei do not show this decay (Fig 1D) [8]. To robustly detect an intensity decay, a 3μm wide subregion is created around the separation line that arises from watershed segmentation (Fig 1D-13). For every pixel of the separation line, a perpendicular intensity profile is measured along the width of the subregion, and the median of these individual intensity profiles is calculated (Fig 1C-14). If the min/max ratio of the median intensity profile is larger than a user-assigned cut-off (typically set at 75%), the split is regarded as incorrect and the two parts of the nucleus are merged. If the min/max ratio is smaller, the split is regarded as being correct and two new nuclear ROIs are generated (Fig 1C-15).

### Validation

Validation of the BleND segmentation algorithm was done by comparing the automatically detected contours (C) with manual delineations of 104 nuclei obtained from three independent observers (ground truth, GT_k_, k = {1,2,3}). To quantify the segmentation performance, two error metrics were used: the average Hausdorff distance (AHD) and non-similarity index (NSI). The AHD is a proxy for the minimal distance between the automatically detected contours C and manually delineated contour GT (Eq 2) [40] and is calculated as follows: for all *p* points of contour C describing nucleus *i*, the minimum Euclidian distance (*d*) to contour GT_k_ is calculated. The average of these distances is the AHD for nuclei *i* with contour C as reference (*h(C*_*i*_, *GT*_*k*,*i*_*)*). Since *h(C*_*i*_, *GT*_*k*,*i*_*)* is not equal to *h(GT*_*k*,*i*_, *C*_*i*_*)*, both are calculated and the maximum of these two values is retained as AHD between contour C_i_ and the k^th^ ground truth for nucleus *i*. The NSI is calculated as the ratio of the non-overlapping area and the sum of the total area enclosed by both contours (C and GT, Eq 3) (Fig 3) [41]. For every nucleus *i*, both error metrics are scaled to a positive control (PC, Eq 4), which is defined as the average error of pairwise comparisons between the three independent GTs (Eq 5). The global error that was used to quantify the actual precision of the automated segmentation is the mean of both scaled parameters (Eq 6).

$$\{\begin{array}{c} h\left(C_{i},GT_{k,i}\right)=\frac{1}{p}\sum_{a\in C_{i}}{\text{min}}_{b\in GT_{k,i}}d\left(a,b\right)\\ {\text{AHD}}_{i}=\frac{1}{3}\sum_{k\in \left\{1,2,3\right\}}\text{max}\left[h\left(C_{i},GT_{k,i}\right),h\left(GT_{k,i},C_{i}\right)\right] \end{array}$$ (2)

$${\text{NSI}}_{i}=\;\frac{1}{3}\sum_{k\in \left\{1,2,3\right\}}1-\frac{\text{Area}\left(C\wedge GT_{k,i}\right)}{\text{Area}\left(C_{i}\right)+\text{Area}\left(GT_{k,i}\right)}$$ (3)

$$\{\begin{array}{c} {\text{AHD}}_{i,scale}=\frac{{\text{AHD}}_{i}}{{\text{AHD}}_{i,PC}}\\ {\text{NSI}}_{i,scale}=\frac{{\text{NSI}}_{i}}{{\text{NSI}}_{i,PC}} \end{array}$$ (4)

$$\{\begin{array}{c} {\text{AHD}}_{i,PC}=\frac{1}{6}\sum_{k\in \left\{1,2,3\right\}}\sum_{l\in \left\{1,2,3\right\}}\text{max}\left[h\left(GT_{k,i},GT_{l,i}\right),h\left(GT_{l,i},GT_{k,i}\right)\right]\\ {\text{NSI}}_{i,PC}=\frac{1}{6}\sum_{k\in \left\{1,2,3\right\}}\sum_{l\in \left\{1,2,3\right\}}1-\frac{\text{Area}\left(GT_{k,i}\wedge GT_{l,i}\right)}{\text{Area}\left(GT_{k,i}\right)+\text{Area}\left(GT_{l,i}\right)} \end{array}$$ (5)

$$Error_{i}=\frac{1}{2}\left({\text{NSI}}_{i,scale}+{\text{AHD}}_{i,scale}\right)$$ (6)

### Data analysis

Classification of the nuclei, identified by the segmentation algorithm, was achieved using a morpho-textural feature set, including all standard ImageJ/FIJI shape attributes (area, perimeter, descriptors of the fitted ellipse, circularity, solidity) as well as curvature and texture descriptors. The curvature of the nuclear boundary (defined by the refined ROI) was depicted as the alteration of the orientation of subsequent edge segments. The total curvature was then calculated as the summation of the absolute values of the first derivatives of these segments. Other features describing the shape of the nuclei are the rotation-invariant elliptic Fourier descriptors (EFD) [42]. The gray-level co-occurrence matrix (GLCM) was calculated to extract features describing the texture of the cell nuclei [43]. The GLCM attributes obtained under different angles (0°, 45°, 90° and 135°) were averaged to obtain rotation invariant parameters describing the texture.

The HDF-NCP data set consisted of 162 dysmorphic nuclei and 831 normal nuclei from which 162 were randomly sampled to obtain a dataset with equal fractions for the two classes. Data analysis was done in R [44]. To explore the data and select the most informative features, principal component analysis (PCA) was performed. Selected features were used for training supervised classification schemes based on linear discriminant analysis (LDA), quadratic discriminant analysis (QDA), regularized discriminant analysis (RDA), mixture discriminant analysis (MDA), naive Bayes (NB), flexible discriminant analysis (FDA), support vector machine (SVM), bagging (BAG), boosting (BOO) and random forest (RF). Different kernels were used in NB and SVM classifiers, whereas different regression methods were used to train FDA classification schemes. For classification, the dataset was split up in a test set (1/3) and training set (2/3) with equal class ratios. Using 10-fold cross-validation in the training set, the classification algorithms were trained and then used on the test set to determine misclassification rate (MCR) and false negative rate (FNR).

## Results

### Automatic segmentation matches manual delineation

To quantify the accuracy of the detection algorithm, the method was compared to three independent GTs of manually delineated HDF-NCP nuclei, using an integrated performance error, based on the average of AHD and NSI as described in the M&M section. The inter-individual variability of the GTs—calculated as the standard deviation of the error scores obtained after pairwise comparison of all GTs—was 7% for all nuclei and 9% for the dysmorphic nuclei only. Using the same error metric, a quantitative comparison was made of the segmentation algorithm using single (n = 16 threshold methods) or two-pass (n = 16 x 16 = 256 threshold combinations) thresholding, with and without contour refinement (Fig 4). On the complete dataset (Fig 4A), a single threshold could not attain the precision of the manual segmentation, since all of the obtained error scores were higher than those observed when comparing the GTs (grey dots). Two-pass thresholding improved the segmentation and resulted in 3 threshold combinations that lied within the GT error range (*i*.*e*., the range of errors obtained by pairwise comparison of individual GT’s, green-coded dots). Contour refinement boosted the performance and reduced the error scores significantly, resulting in 100 threshold combinations (39% of the 256 combinations) that resulted in values within this error range. The fact that multiple threshold combinations yielded errors within the GT error range indicates that the BleND algorithm attains the precision of manual delineation. For segmentation of dysmorphic nuclei, both 2-pass thresholding (P = 0.04756; Mann—Whitney U test, one-sided) and contour refinement (P = 0.004939; Mann—Whitney U test, one-sided) enhanced segmentation error scores in a statistically significant manner (Fig 4B). Some threshold methods served better as global method (e.g. Max Entropy) or as local method (e.g. Li), whereas others performed poorly throughout (e.g. Shanbhag).

### BleND accurately delineates nuclei in a variety of cell types and data sets

To assess the generic value of the segmentation algorithm, BleND was also tested on images of a variety of cell types with aberrant nuclei such as HDF-HGPS, HDF-NULL, HeLa-ZKO, HT-LKO and mouse primary hippocampal neurons acquired with different imaging modalities at 40x or 60x magnification (Fig 5A). Lower magnifications were not considered since the main objective of BleND is to detect subtle deviations of nuclear shapes, which are not clearly visible at low resolution. All cells are characterised by dysmorphic nuclei, but they differ strongly in shape and texture: nuclei of HDF-NULL and HT-LKO cells are severely deformed and often show an intensity gradient due to chromatin ruffling, whereas HeLa-ZKO cells and primary hippocampal neurons have nuclei with small blebs and especially the latter show high intranuclear intensity spots (chromocenters). For all image types, suitable segmentation settings could be defined (Fig 5A).

### Comparison with nuclear segmentation methods

Next, error scores of the BleND algorithm were compared with other state-of-the-art algorithms used for the analysis of nuclear morphology [12,45]. For this, a rough segmentation was performed using the 2-pass thresholding algorithm implemented in BleND, after which different refinement steps were compared: dynamic programming (BleND), level set (LS) active contour (Fiji) [23,45] and gradient vector flow (GVF) active contour (Matlab^®^) [12,13,46]. To allow accurate delineations of blebs and crevices, curvature penalty weights were decreased for both LS and GVF active contour. The results are represented in Table 1. GVF active contour and BleND showed a similar error and generated segmentations that attain the precision of the GT for normal nuclei. On the other hand, for dysmorphic nuclei, BleND was the only algorithm that could attain GT precision (Fig 5B). In general, the LS active contour algorithm resulted in inaccurate segmentations of nuclei with lower intensity or intensity gradients (Fig 5B).

**Table 1:** Performance errors of automatic segmentation methods.

Method	Class	Error
BleND*	Normal	0.935±0.222
	Dysmorphic	0.936±0.131
LS**	Normal	1.607±0.861
	Dysmorphic	2.678±2.243
GVF***	Normal	1.022±0.345
	Dysmorphic	1.153±0.386

*Threshold settings: Global = Triangle, Local = Mean

**LS = Level Set Active Contour: Advection 2.20, Curvature 0.10, Grayscale Tolerance 0.01, Convergence 0.0030

***GVF = Gradient Vector Flow Active Contour: Iterations 400, Tension or alpha 0, Rigidity or beta 0, External force or kappa, 30.

### Clustering of normal and dysmorphic nuclei

After validation of the algorithm, morpho-textural features were extracted from the segmented nuclei of HDF-NCP (Fig 6A). Hierarchical clustering of the normalized data based on Manhattan distance and Ward’s clustering method, identified two major clusters, largely corresponding to the normal and dysmorphic nuclei, with ~ 89% correspondence to the manually assigned classes (Fig 6B). Visual inspection of incorrectly clustered nuclei revealed that their classification is often dubious, due to the presence of rough boundaries (in normal nuclei) or absence of overt blebs (for aberrant nuclei) (Fig 6C). The heatmap revealed a higher correlation of several shape parameters and the lack of correlation with textural features, as could be expected for this specific dataset since the HDF-NCP nuclei do not show any discriminating intensity-based characteristics. Indeed, EFD, curvature, solidity and circularity features comprised the most relevant information. This qualitative evaluation was confirmed by PCA, which revealed that the first principal component (PC1), explaining 29.25% of the variance within the dataset, contained no texture feature and determined the strongest direction of class separation. PC1 was defined by the EFD, curvature, solidity and circularity features (Fig 6D and 6E). The performance of hierarchical clustering could however not be improved significantly by using a reduced feature subset from PC1 (absolute correlation with PC1 larger than 0.7) (data not shown). As could be expected, EFD parameters dominated both clustering and PCA. Tracing the values of the summed EFD back to the segmented nuclei revealed a strong correlation between the severity of the shape alterations and the EFD value (Fig 7A). However, the summed EFD score by itself was not enough to distinguish all nuclei, since there was an overlap in the 0.4–0.5 range between normal and dysmorphic nuclei, thus calling for integration of other morphological parameters. In the NCP dataset, textural parameters had little impact on the classification result, but there were conditions were texture did significantly add to the discriminatory power. Indeed, in many HDF-NULL cells, nuclei are not only dysmorphic, but also show local chromatin ruffling. This feature could effectively be picked up by textural features and allowed discrimination of morphologically similar nuclei (Fig 7B).

### Supervised classification enables robust detection of dysmorphic nuclei

Using the selected feature set, a classifier was built for predicting nuclear dysmorphy. Various classification algorithms were assessed; their optimal MCR and the FNR are listed in Table 2. A support vector machine with a radial basis function (i.e. Gaussian) kernel yielded the best FNR, whereas a random forest classifier (300 trees, 5 features) had the best MCR on a training set through 10-fold cross-validation. On an independent test set, the support vector algorithm attained the best results with an MCR of 4.65% and a FNR of 0.92%. Because of their high performance, trainable SVM and RF classifiers (WEKA library [47]) were integrated in BleND. Herein, segmented nuclei can be assigned with a user-defined label through a graphical user interface. After manually categorizing a set of nuclei, a classifier is built and used to predict the classes for a larger set of segmented nuclei. Predictions can be improved by iterative addition of new manually assigned classes and classifier building (S1 Fig).

**Table 2:** Classification performance of different classifiers* on a training and test set using 10-fold cross-validation.

	LDA	QDA	RDA	MDA	FDA	NB	SVM	BAG	BOO	RF
**TRAIN MCR**	10.6	9.3	9.7	7.9	9.25	10.6	7.9	7.8	9.3	6.0
**TRAIN FNR**	7.9	0.9	7.0	4.1	4.2	5.1	0.9	2.3	4.6	1.9
**TEST MCR**	13.9	11.1	15.7	11.1	10.2	11.1	4.6	12.0	13.9	10.2
**TEST FNR**	10.2	4.6	13.0	7.4	5.6	6.5	0.9	5.6	7.4	4.6

*Settings: RDA: lambda 0.8997867, MDA: 3 subclasses, NB: Epanechnikov, SVM: Gaussian, BAG: 50 trees, BOO: 4561 iterations, RF: 500 trees, 3 features)

## Discussion

Dysmorphic nuclei are characteristic for a wide range of pathologies such as cancer, viral infections and nuclear envelopathies. Automated recognition and analysis of these nuclei may enhance the efficiency of cell-based microscopy experiments aimed at unraveling mechanisms underlying pathology. To this end, we wrote an algorithm that is tailored towards segmentation of dysmorphic nuclei and can be used for a wide variety of cell types acquired with different image modalities. Based on an integrated error score, we have shown that BleND attained a precision that matched the ground truth, when taking into account an inter-individual variability of 7%. The algorithm was further used to build a classifier that accurately predicts whether a nucleus is normal or dysmorphic.

Crevices and blebs that define dysmorphic nuclei entail major challenges due to their possible small size and lower intensity. Since there is no prior knowledge about the location, shape or intensity other algorithms described in literature are less suitable for this purpose. Segmentation algorithms relying on shape-based seed detection are not applicable to detect dysmorphic nuclei, since the shape of these nuclei strongly deviates from the normal convex shape [16,17]. Other algorithms use intensity information for the segmentation of the nuclei. However, local intensity minima in blebs can negatively influence the result of level sets-based methods as proven when comparing to BleND [48].

An algorithm that is optimised for the detection of nuclei with small aberrations has been described and is based on an GVF active contour algorithm [12]. As shown, error scores of this algorithm were similar to those of BleND for normal nuclei, but only BleND could attain the precision of manual delineations for dysmorphic nuclei. In line with the results of Driscoll *et al*, we found that curvature and solidity are good predictors of nuclear blebbing [12]. However, we now also show that EFD parameters are stronger correlates of nuclear dysmorphy and that the sum of these features correlates strongly with the severity of the deformation. In addition, BleND offers an alternative approach based on a simple DAPI staining rather than an immunofluorescence labelling of lamin A/C, making it more amenable for rapid, routine screening and multiplexing.

We have supported BleND with a framework for quantitative estimation of segmentation performance. The scoring system is based on scaled error metrics that describe the difference between the automated segmentation and user defined ground truths, and makes it possible to select the best threshold combination for the image data sets at hand. Our results demonstrate that a combination of global and local thresholding outperforms a single thresholding step. For contour refinement, we make use of a dynamic programming approach that is preceded by a straightening step and edge enhancement (derivative) of the initial contour. Other dynamic programming alternatives described in literature use polar transformation instead of bilinear interpolation for straightening of the edge [17,18]. This requires a centre and contour point as well as a mean radius to be defined in order to transform Cartesian into polar coordinates. Since dysmorphic nuclei are characterized by their non-circularity, a polar transformation seems unfit. Normalisation of the columns equalizes the weight of all pixel values, causing the original lower intensities of edges in blebs or crevices to have an equal influence on the average path strength. In addition, the algorithm allows propagation angles greater than 45° to accurately describe crevices surrounding nuclear blebs. Segmentation results using the contour refinement algorithm were significantly better than those using only a thresholding step.

Morpho-textural features were extracted from the segmented nuclei and used for supervised classification with an accuracy up to 95%. Classification of the HDF-NCP nuclei was mainly determined by features that describe the shape of the nuclei such as curvature and the EFD descriptors. This is not surprising, as this type of cells does not show major textural alterations. However, other cell types such as HDF-NULL cells do show biologically relevant intensity variations (reflecting chromatin ruffling [21]) that may need to be discriminated. We showed that for similarly shaped nuclei, texture metrics such as entropy can discriminate chromatin ruffling. Thus, including these textural features makes BleND applicable to a broad range of cell types showing nuclear alterations.

The automated recognition is perfectly suited to be implemented in high-content perturbation screens that score nuclear shape changes associated with knockdown of specific genes [49,50] or treatment with chemical compounds [51]. A next logical step would be to integrate this automated recognition algorithm in an intelligent imaging workflow [52–54]. During live cell imaging, relevant events may be missed, since the observer manually has to define a region of interest before starting the experiment and the time resolution per well or spot is limited [55]. However, when the scope of the experiment can be limited to only those nuclei of interest (*in casu*, dysmorphic nuclei), the efficiency may be significantly increased. Feedback regulation between the microscope and the algorithm can result in automatic recognition of dysmorphic nuclei and subsequent initiation of an appropriate acquisition. A first step towards an integrated, broadly applicable intelligent imaging workflow, is the implementation of an iterative machine learning scheme in which a classifier can be trained on the fly, i.e. whilst images are being acquired [50]. As proof of principle, such an iterative learning process was integrated in BleND, resulting in classification scores similar to those of the independent data analysis that was performed. This workflow can be the starting point for high-resolution follow-up of more deformable nuclei [56], or even more complex imaging schemes such as selective, functional imaging (FRET, FCS, FRAP…) of dysmorphic nuclei, as has been demonstrated for mitotic phenotypes [57].

In conclusion, the proposed method can accelerate both fundamental research as well as diagnostics of the broad range of pathologies that are linked to nuclear dysmorphy.

## Supporting Information

S1 Fig Improved predictions by iterative training of implemented classifier. The misclassification rate (MCR) declines after iterative training of the implemented classification algorithm through a graphical user interface. In this example, a random forest classifier was used (100 trees, 7 features).
(TIFF)
