# Correction: Accurate Detection of Dysmorphic Nuclei Using Dynamic Programming and Supervised Classification

**DOI:** 10.1371/journal.pone.0173701

**Published:** 2017-03-06

**Authors:** The PLOS ONE Staff

The numbers “17020013” are superimposed over Fig 1C. Please see the corrected Fig 1 here. The publisher apologizes for the error.

**Fig 1:**
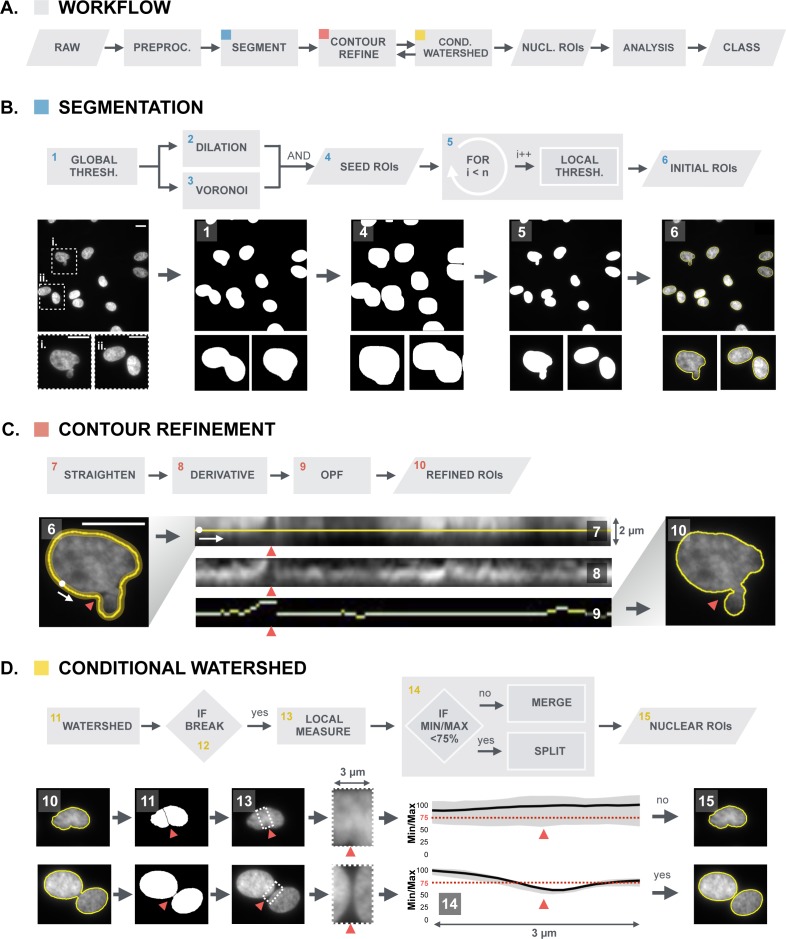
Overview of the BleND segmentation algorithm. (A) Workflow of the algorithm built on (B) intensity-based segmentation, (C) contour refinement, and (D) conditional watershed; (B) The segmentation process is implemented as a two-pass thresholding algorithm that generates “initial ROIs” of nuclei in the preprocessed image (i: dysmorphic nucleus, ii: two juxtaposed normal nuclei). A global thresholding is performed on the image, which creates a binary mask (1). The objects identified herein are dilated by 3 μm and combined (Boolean AND operation) with a Voronoi tessellation mask to ascertain that the dilated objects do not fuse. For each resulting “seed ROI” (4), a local threshold (5) is determined yielding an initial nuclear ROI (6) that is more accurate than the seed ROI (note the improved segmentation for the dysmorphic and juxtaposed nuclei); (C) In the subsequent contour refinement procedure, the initial ROI is used (6) to straighten a 2μm wide region along the nuclear periphery (white dot indicates the point where the contour was opened and the white arrow indicates the direction of the straightening) (7). In this rectangular representation, the edge of the nucleus is enhanced by convolution with a vertical Sobel kernel (8). Then, an optimal path finding (OPF) algorithm determines the path with the highest path strength (9). The OPF algorithm effectively detects crevices surrounding nuclear blebs (red arrowhead). The contour of the nucleus is then reconstructed to generate a “refined ROI” and this process is repeated until the optimal path no longer changes (10); (D) To segment neighboring nuclei that could not be separated in the previous steps, a conditional watershed was implemented in which correct and incorrect splits were discriminated based on a size criterion and an intensity drop along the separation line (red arrowhead). This intensity drop is calculated as a median intensity profile perpendicular to the separation line (13). The user defines a threshold for the acquired intensity drop. In this example, the threshold is set at 0.75. If there is an intensity drop in the median profile of less than 25%—Min/Max intensity ratio above the 75% (dotted red) line (14)—the split is regarded as incorrect and the two parts of the nucleus are merged (15). If the drop is bigger, the split is regarded as being correct and it is retained to generate new nuclear ROIs.
