# Supplementary figures and images for: Accurate Detection of Dysmorphic Nuclei Using Dynamic Programming and Supervised Classification
Source: PLoS One. 2017 Jan 26;12(1):e0170688. doi: 10.1371/journal.pone.0170688 (PMC5268651; doi:10.1371/journal.pone.0170688)

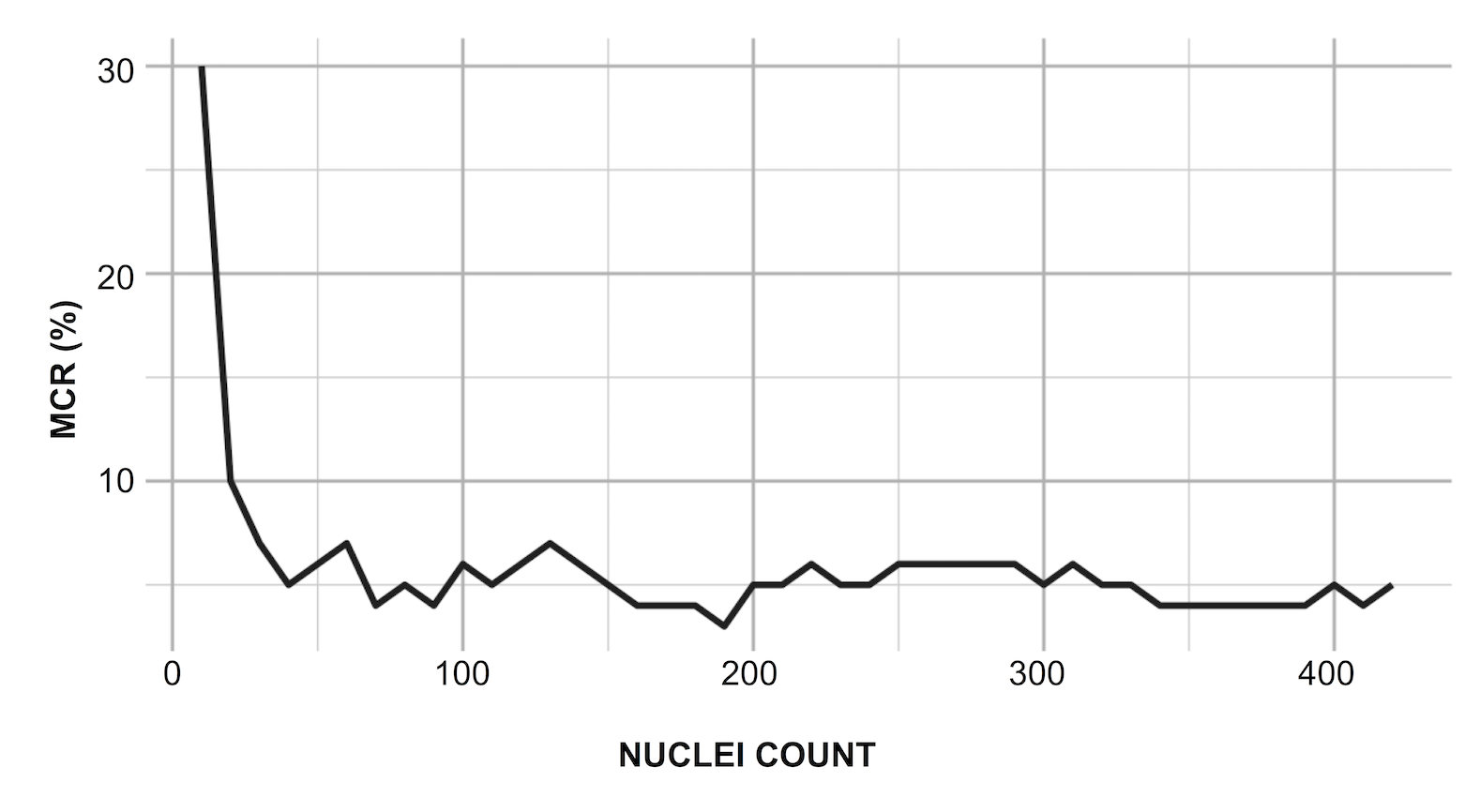

Supplement: S1 Fig — The misclassification rate (MCR) declines after iterative training of the implemented classification algorithm through a graphical user interface. In this example, a random forest classifier was used (100 trees, 7 features). (TIFF) [file pone.0170688.s001.tiff]
